# Arrhythmia risk stratification late after Tetralogy of Fallot repair with pulmonary regurgitation

**DOI:** 10.1016/j.ijcchd.2022.100409

**Published:** 2022-07-13

**Authors:** Maria Drakopoulou, Evangelia Nyktari, Anastasios Apostolos, Skevos Sideris, Athanasios Kordalis, Georgios Oikonomou, Konstantinos Tsioufis, Konstantinos Toutouzas, Konstantinos Gatzoulis

**Affiliations:** a First Department of Cardiology, Medical School, National and Kapodistrian University of Athens, Hippokration General Hospital, Athens, Greece; b Department of Cardiology, Onassis Cardiac Surgery Center, Athens, Greece; c Department of Cardiology, Hippokration General Hospital

**Keywords:** Tetralogy of fallot, Ventricular tachycardia, Risk stratification

## Abstract

Among the most feared sequelae of repaired Tetralogy of Fallot (TOF) are ventricular arrhythmias and sudden cardiac death (SCD) [1]. The presence of pulmonary regurgitation as a potential risk factor for arrhythmias has sparked interest in a more aggressive strategy for reoperation in TOF patients and chronic pulmonary regurgitation. Although this strategy was initially suggested to be protective against the development of ventricular arrhythmias, timely pulmonary valve replacement (PVR) alone, does not appear to abort the SCD risk as myocardial fibrosis, a clear arrhythmic substrate remains. In this case, we present arrhythmia risk stratification and management of a patient with repaired ToF and concomitant pulmonary regurgitation.

A 46-year-old female patient with history of ToF repair (transannular patch and augmentation of right ventricular outflow tract) presented with episodes of atypical chest pain (located at the substernal area, lasting for few seconds, not related to exercise). The patient had no restriction in exercise capacity. The patient was on sinus rhythm with RBBB and QRS duration of 140 ms. The signal-averaged electrocardiogram (SAECG) was abnormal (2 out of 3 criteria). Transthoracic echocardiography showed mildly dilated right ventricle (RV) with preserved systolic function and moderate pulmonary stenosis with moderate-to severe regurgitation (Fig. 1A–C). The left ventricle was mildly dilated with preserved ejection fraction and moderate aortic valve regurgitation (Fig. 1D–F). A sizeable, aneurysmatic-dyskinetic region (∼28mm) was observed in RV outflow tract by cardiac magnetic resonance imaging (MRI) (Fig. 2A–C). Myocardial late gadolinium enhancement (LGE) was observed in all surgically affected areas and the RV outflow tract dyskinetic region (Fig. 2D). Coronary angiogram showed non-obstructed coronary arteries. Holter monitoring revealed episodes of complex QRS tachycardia at 110bpm (duration∼10 sec). During the electrophysiological study, syncopal sustained ventricular tachycardia (VT) was induced (CL:225 ms, inferior axis, LBBB) and the patient recovered with defibrillation.

**Fig. 1:**
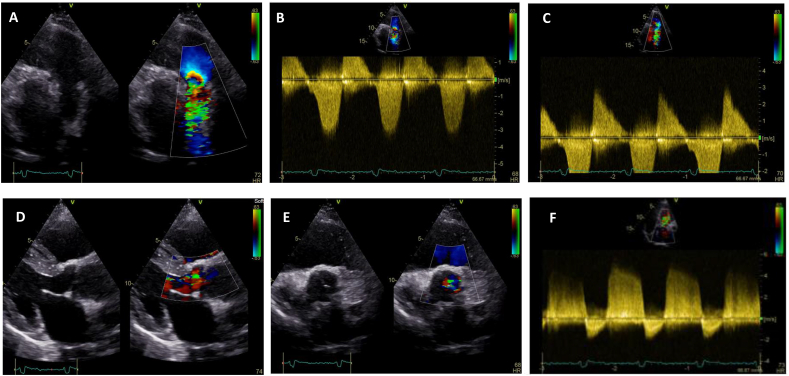
Transthoracic echocardiography showing moderate pulmonary stenosis (Vmax: 3.5 m/s, MG: 28 mmHg, PG:45 mmHg) with moderate to severe regurgitation (PHT∼170 ms) (A,B,C) and moderate aortic valve regurgitation due to mild aortic root dilation (40mm) with a central jet (PHT∼460 ms) (D,E,F).

**Fig. 2:**
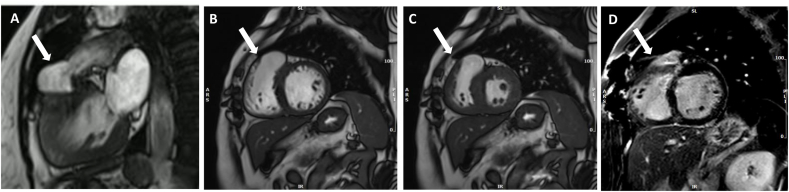
Cardiac magnetic resonance imaging (MRI) showing a showing a sizeable, aneurysmatic region of 28mm at the right ventricular outflow tract (A: sagittal plane, B, C: coronal plane). Myocardial late gadolinium enhancement (LGE) was observed in all surgically affected areas and the RV outflow tract dyskinetic region (D).

Overall, symptoms of chest discomfort were attributed to episodes of non-sustained VT rather than pulmonary regurgitation. Based on the risk score previously proposed by Khairy et al., the patient was classified in the high-risk population (17.5% estimated annualized rate of appropriate shocks) [11]. Thus, there was a decision to proceed to the implantation of an MRI-conditional implantable cardioverter defibrillator system. The patient was scheduled to be re-evaluated for potential surgery in 1 year. At 3-month follow-up the patient was asymptomatic - free of chest pain.

In repaired ToF, the presence of chronic pulmonary regurgitation renders arrhythmia risk stratification challenging. Right ventricular volume overload induced by pulmonary regurgitation may act as an important trigger factorfor arrhythmias due to increased RV end-diastolic pressure, progressive myocardial remodeling, and fibrosis. Based on recent guidelines, pulmonary valve replacemnet (PVR) is indicated in symptomatic patients with severe pulmonary regurgitation and/or at least moderate RV outflow tract obstruction [2]. In asymptomatic patients with severe pulmonary regurgitation though, indication and timing for reintervention remains uncertain. Several factors are integrated in decision-making processes such as decreased exercise capacity, preoperative RV volumes, progressive RV systolic dysfunction, and progression of tricuspid regurgitation to at least moderate. Still however, current guidelines do not consider ventricular arrhythmias as a factor for PVR, as there is no robust evidence that PVR reduces the risk of subsequent ventricular arrhythmias [2].

Because sudden cardiac death (SCD) prevention and pulmonary regurgitation management are two major issues in adult patients with TOF, the impact of PVR on arrhythmia propensity and electrical remodeling is of particular importance. The degree to which RV remodeling after PVR may reduce the arrhythmogenic substrate, however, is unknown. Moreover, most available studies included a limited number of patients and there is no published data demonstrating a clear survival benefit from PVR (Table 1) [[3], [4], [5], [6], [7], [8], [9]]. Therrien et al. demonstrated for the first time that PVR late after TOF repair has a stabilizing effect on QRS duration and, in conjunction with intraoperative cryoablation, leads to a decrease in the incidence of preexisting atrial and ventricular tachyarrhythmia [3]. A recent large propensity score–adjusted analysis showed that PVR was not associated with a reduced rate of a composite outcome of death or VT over a mean follow up of 5.3 years [7]. In a nationwide multicenter cohort of TOF patients with an ICD, Bessière et al., observed a significant decrease of appropriate ICD therapies burden after PVR [8]. Pulmonary valve replacement before implantation was also associated with a lower risk of appropriate ICD therapy in primary prevention patients.

**Table 1:** Studies evaluating the impact of PVR in arrhythmias and SCD.

Study	Year	Study Population	Aim	Findings
Therrien J et al. [3]	2001	70 patients who underwent PVR late after repair of TOF	Examine the effects PVR on 1) ECG markers predictive of monomorphic VT and SCD and 2) incidence of sustained atrial flutter/fibrillation and/or monomorphic VT.	QRS duration remained unchanged in the study group (p = 0.46). At a mean follow-up of 4.7 years, the incidence of ventricular tachycardia diminished from 22% to 9% (p < 0.001). Intraoperative ablation prevented recurrence of preexisting tachyarrhythmia.
Karamlou T et al. [4]	2006	249 patients who underwent PVR late after repair of TOF	Determine 1) risk factors for recurrent arrhythmia and death after reoperation and 2) whether arrhythmia surgery after reoperation decreases the risk of recurrent arrhythmia.	Risk factors for post-reoperative recurrent arrhythmia were longer QRS duration and not having PVR.
Gengsakul A et al. [5]	2007	82 patients with PVR after late after ToF and control non-PVR patients	Determine the impact of PVR on clinical outcomes	Symptoms and functional class improved in PVR patients with no change in the non-PVR patients (p < 0.001). QRS duration change was not significantly different between PVR and non-PVR patients (p = 0.48). A significant impact on arrhythmia was not detected.
Harrild DM et al. [6]	2009	98 patients who underwent PVR late after repair of TOF	Determine the incidence of death and VT in TOF after PVR and to test the hypothesis that PVR leads to improvement in these outcomes.	Late PVR for symptomatic pulmonary regurgitation/RV dilation did not reduce the incidence of VT or death
Bokma JP et al. [7]	2018	249 patients after repair of TOF	Determine the association of PVR with death and sustained VT in patients with repaired TOF.	PVR was not associated with a reduced rate of death and sustained VT at an average follow-up of 5.3 years. Late mortality in patients at risk for recurrent VT and supraventricular tachycardia, but recurrent VT is uncommon.
Bessiere et al. [8]	2021	165 patients after repair of TOF with ICD	Assess the impact of PVR on ventricular arrhythmia burden in TOF patients with ICD.	The burden of appropriate ICD therapies was significantly lower after PVR (HR: 0.21; 95% confidence interval [CI]: 0.08 to 0.56; p < 0.002).
Tal Geva et al. [9]	2018	452 patients who underwent PVR late after repair of TOF	Identify pre-PVR predictors for post-PVR sustained VT and SCD.	An older age at PVR and pre-PVR right ventricular hypertrophy and dysfunction were predictive of a shorter time to postoperative death and sustained VT.

A comprehensive, multidisciplinary, and tailored preoperative approach should be applied, to choose the optimal timing of operation and the extent of preoperative arrhythmic risk stratification. ECG, SAECG, Holter monitoring,cardiopulmoanry exercise test (CPET) and cardiac imaging should be performed to all ToF patients with pulmonary regurgitation; high-risk patients should undergo electrophysiology study [1,10]. Abnormal findings in the electrophysiology study, preoperative syncope and VT are the main indications for ICD placement or surgical cryoablation. The exact locations, where cryoablation should be performed, remain under investigation.

In conclusion, the management of repaired ToF patients with chronic pulmonary regurgitation remains challenging and clearly requires tertiary expertise. The optimal time for arrhythmic risk stratification and intervention cannot be clarified precisely with the current literature and existing studies. Long-term, prospective studies assessing patient selection for ICD implantation for primary prevention of SCD and or optimal time for PVR may shed additional light and are clearly warranted.

## Patient's consent

Patient has granted consent to publishing.

## Declaration of competing interest

None.
